# A Bispecific Protein Capable of Engaging CTLA-4 and MHCII Protects Non-Obese Diabetic Mice from Autoimmune Diabetes

**DOI:** 10.1371/journal.pone.0063530

**Published:** 2013-05-21

**Authors:** Hongmei Zhao, Jozsef Karman, Ji-Lei Jiang, Jinhua Zhang, Nathan Gumlaw, John Lydon, Qun Zhou, Huawei Qiu, Canwen Jiang, Seng H. Cheng, Yunxiang Zhu

**Affiliations:** Genzyme, a Sanofi Company, Framingham, Massachusetts, United States of America; University of Miami, United States of America

## Abstract

Crosslinking ligand-engaged cytotoxic T lymphocyte antigen-4 (CTLA-4) to the T cell receptor (TCR) with a bispecific fusion protein (BsB) comprised of a mutant mouse CD80 and lymphocyte activation antigen-3 (LAG-3) has been shown to attenuate TCR signaling and to direct T-cell differentiation toward Foxp3^+^ regulatory T cells (Tregs) in an allogenic mixed lymphocyte reaction (MLR). Here, we show that antigen-specific Tregs can also be induced in an antigen-specific setting in vitro. Treatment of non-obese diabetic (NOD) female mice between 9–12 weeks of age with a short course of BsB elicited a transient increase of Tregs in the blood and moderately delayed the onset of autoimmune type 1 diabetes (T1D). However, a longer course of treatment (10 weeks) of 4–13 weeks-old female NOD animals with BsB significantly delayed the onset of disease or protected animals from developing diabetes, with only 13% of treated animals developing diabetes by 35 weeks of age compared to 80% of the animals in the control group. Histopathological analysis of the pancreata of the BsB-treated mice that remained non-diabetic revealed the preservation of insulin-producing β-cells despite the presence of different degrees of insulitis. Thus, a bifunctional protein capable of engaging CTLA-4 and MHCII and indirectly co-ligating CTLA-4 to the TCR protected NOD mice from developing T1D.

## Introduction

Cytotoxic T lymphocyte antigen-4 (CTLA-4), also known as CD152, is a negative regulator of the T-cell response. This protein has been shown to play an important role in the maintenance of T-cell homeostasis and in the induction of self-tolerance [1]–[5]. Mice deficient in CTLA-4 develop multi-organ autoimmune disease and typically succumb to the ailment by 4 weeks of age [6], [7]. The molecular mechanisms through which CTLA-4 modulates T-cell activity are multifaceted and are thought to occur either intrinsically on conventional T cells or extrinsically through Tregs [8]–[10]. These mechanisms include competing with CD28 for ligand binding [11], inducing the production of the tolerogenic enzyme indoleamine 2,3 dioxygenase in antigen presenting cells (APCs) [12], [13], and displacing CD28 from the immunological synapse [14]. The engagement of CTLA-4 by its ligands (CD80/86) on APCs also stimulates the recruitment of the phosphatases SHP-1 [15] and PP2A [16], [17] to the vicinity of the TCR on T cells undergoing activation. The consequent dephosphorylation of the key signaling molecules associated with the TCR results in the termination of T cell activation [18]. Moreover, interventions that promote the early engagement of CTLA-4 with its ligands and crosslinking to the TCR result in the premature dampening of key signaling signatures and the consequent inhibition of T-cell activation, leading to T-cell hyporesponsiveness or anergy [18]–[21].

To promote the crosslinking of CTLA-4 to the TCR during the early phase of T-cell activation, we had previously reported on the merits of a bispecific fusion protein (designated as BsB hereafter in the paper) comprised of a mutant CD80 (CD80w88a) and the lymphocyte activation gene-3 (LAG-3) as a proof-of-concept molecule. BsB was designed to concurrently engage CTLA-4 and MHCII in the immune synapse and thereby indirectly crosslink CTLA-4 to the TCR via the cognate pairing of MHCII with the TCR [22]. In an allogenic MLR, BsB was shown to be effective at inhibiting T cell activation. Interestingly, BsB also induced the production of IL-10 and TGF-β and promoted the differentiation of T cells undergoing activation to Foxp3^+^ Tregs. Therefore, each of these components may provide anti-inflammatory benefits for immunomodulation separately or in combination. IL-10 can exert broad immunosuppressive properties through its ability to control the activation of macrophages and dendritic cells (DCs), as well as to self-regulate Th1 cells [23]. TGF-β can act as an inhibitor of T-cell differentiation [24], macrophage activation [25], [26] and dendritic cell maturation [27]. In addition to their anti-inflammatory functions, IL-10 and TGF-β can purportedly influence Treg function. For example, IL-10 has been shown to induce IL-10-producing Tr1 cells [28] and to act on Foxp3^+^ Tregs to maintain the expression of Foxp3 and thereby propagate the suppressive function of the Tregs [29]. Similarly, TGF-β has been reported to be necessary for the induction of Tregs [30], [31] and in maintaining their suppressive function by promoting Foxp3 expression [32].

Tregs are a functionally distinct subpopulation of T cells that are capable of controlling the immune responses to self and non-self antigens. A deficiency of Tregs results in a heightened immune response and often the presentation of autoimmune diseases [33]. Extensive research has established a role for these specialized T cells in controlling all aspects of immune responses. In particular, there is evidence that Tregs can engender self-tolerance. These findings suggest that agents capable of boosting the in situ production of Tregs or the adoptive transfer of Tregs could be deployed to treat autoimmune diseases. Indeed, Treg-based therapies using freshly isolated or ex vivo-expanded Tregs have been shown to be effective in treating animal models of type 1 diabetes (T1D) [34], [35] and graft-versus-host disease [36]–[38]. In lieu of isolating and expanding Foxp3^+^CD4^+^CD25^+^ Tregs (often designated as natural Tregs or nTregs) from peripheral blood or lymph nodes, Tregs can be induced from naïve CD4^+^CD25^−^ T cells in the context of TCR activation and in the concomitant presence of TGF-β and IL-2. These Tregs are often referred to as adaptive Tregs (aTregs) or induced Tregs (iTregs). They are also Foxp3 positive and purportedly exhibit equally potent suppressive functions compared with nTregs [30], [31], [39]. Adoptive transfers of aTregs/iTregs have been shown to be effective in conferring protection against autoimmune disease in an animal model of collagen-induced arthritis [40]. However, it is becoming evident that antigen-specific Tregs have a significantly higher therapeutic potential than polyclonal Tregs with a pan-TCR repertoire [34], [35], [41] and that antigen-specific Tregs have a potentially lower risk of the side effect of pan-immune suppression. For this reason, we sought to evaluate the capabilities of BsB to produce antigen-specific Tregs in an antigen-specific T-cell activation setting in vitro. Moreover, we tested BsB’s potential to treat autoimmune diabetes in the NOD mouse.

T1D is an autoimmune disease that is caused by the tissue-specific destruction of insulin-producing pancreatic β-cells with the consequent development of hyperglycemia. NOD mice (female mice in particular) spontaneously develop autoreactive T cells towards islet-specific self-antigens (e.g., insulin and glutamic acid decarboxylase 65). In concert with other inflammatory cells, these autoreactive T cells begin to accumulate around the islets (peri-insulitis) between 3 and 4 weeks of age, followed by progressive infiltration of the islets (insulitis) by 9 weeks of age, and spontaneous onset of diabetes between 12 and 35 weeks [42]. NOD mice share many characteristics with T1D in human subjects, such as the production of pancreas-specific autoantibodies and the activation of autoreactive CD4^+^ and CD8^+^ T cells. The susceptibility of these mice to autoimmunity, as in humans, is influenced by genes for the major histocompatibility complex (MHC), CTLA-4, and LAG-3. NOD mice harbor a unique MHC haplotype (H-2^g7^) that reportedly confers the highest risk for disease susceptibility [43], [44]. A CTLA-4 polymorphism has also been noted in NOD mice [45] and in humans [46], and a LAG-3 deficiency on the NOD background accelerates T1D onset with 100% penetrance [47]. Because BsB engages all of these targets, for proof-of-concept, we elected to initially test the therapeutic merits of BsB in this murine model of T1D.

## Results

### BsB Directs the Differentiation of OT-II T Cells into Antigen-specific Tregs

Tregs have shown considerable therapeutic potential in modulating the disease manifestations in several animal models of autoimmune diseases. However, the importance of the specificity of the induced Tregs against the relevant antigens has been highlighted. Non-antigen-specific Tregs that will not be activated against particular autoantigens in the context of autoantigen-specific reactive T cells are presumably not functionally immunosuppressive. Hence, approaches that facilitate the generation of large numbers of antigen-specific Tregs are highly desirable for treating these ailments. Moreover, strategies that facilitate the de novo induction of antigen-specific Tregs in situ (e.g., in the islets of the pancreas for T1D or in the lamina propria for ulcerative colitis or Crohn’s disease) are preferred over the use of adoptive transfer of in vitro-differentiated or in vitro-expanded Tregs.

**Figure 1:**
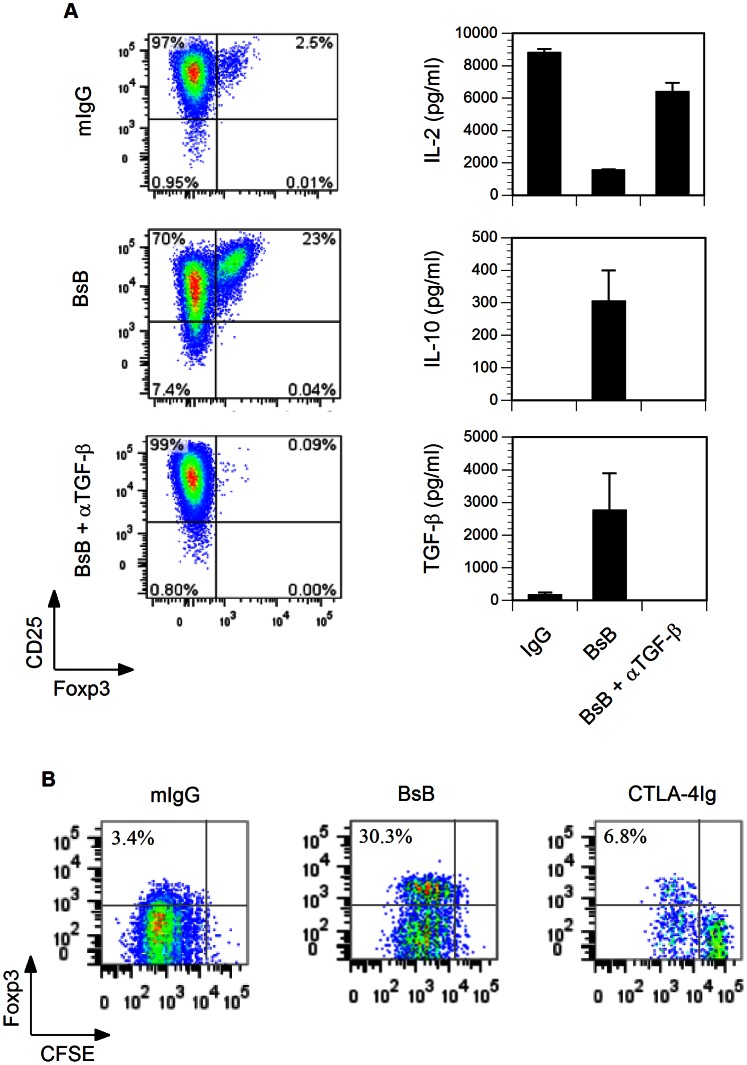
BsB-mediated induction of antigen-specific Tregs in vitro. (A) The in vitro induction of Ova_233–339_-specific Tregs. Naïve OT-II T cells were mixed with LPS-activated and irradiated syngeneic APCs in the presence of 0.5 µg/ml Ova_233–239_ peptide. Control mIgG2a, BsB, and BsB plus an anti-TGB-β antibody (αTGF-β) were then added and tested as indicated (left panels). The cells were cultured for 5 days and then labeled with anti-CD25 and anti-Foxp3 antibodies before being analyzed by flow cytometry. IL-2, IL-10 and TGF-β levels in the culture media were assayed by ELISA. (B) The monitoring of induced Tregs proliferation. The studies were conducted as in (A) except naïve OT-II T cells were pre-labeled with CFSE before being mixed with APCs. The cells were gated on Foxp3 and CFSE fluorescent channels.

Our recent finding that a bifunctional fusion protein comprised of CD80wa and LAG3 (BsB) designed to crosslink CTLA-4 to the TCR (via MHCII) can induce the production of Foxp3^+^ Tregs in an allogenic MLR led us to examine the potential of this protein to elicit the production of antigen-specific Tregs. To investigate this prospect, naïve OT-II T cells were purified from transgenic mice harboring transgenes encoding the TCR (α- and β-subunits) specific for a chicken ovalbumin peptide (323–339) [48] and mixed with syngeneic APCs in the presence of Ova_323–339_. After 5 days of culture, significantly larger numbers of Foxp3^+^ Tregs were detected in the OT-II T cell cultures that had been treated with BsB (Fig. 1A, middle left panel) than in those that were treated with the mouse IgG control (Fig. 1A, upper left panel) or with CTLA-4Ig (data not shown). This induction of Tregs was inhibited by the inclusion of the anti-TGF-β antibody in the cultures, suggesting that the differentiation was mediated in an autocrine or paracrine manner by the endogenously produced TGF-β. The levels of IL-2 were decreased, whereas the levels of IL-10 and TGF-β were increased in the media of the BsB-treated cells (Fig. 1A, right panels).

To monitor the proliferative activity of the induced Tregs, OT-II cells were preloaded with the fluorescent tracer carboxyfluorescein succinimidyl ester (CFSE). As shown in Fig. 1B, BsB-induced Foxp3^+^ Tregs were determined to be proliferative as indicated by a dilution of the CFSE signal. As expected, the addition of CTLA-4Ig, a co-stimulatory blocker, reduced T cell proliferation. Hence, BsB was able to inhibit T cell activation and induce the production of Tregs in both an allogenic MLR and an antigen-specific setting in vitro. Since the OT-II T cells lacked a Foxp3-driven reporter gene such as GFP, antigen-specific OT-II Tregs could not be sorted to homogeneity for the functional suppression assay. However, based on the observed expression of Foxp3, and production of IL-10 and TGF-β, we speculate that the OT-II Tregs were functionally suppressive like the Tregs we generated previously in a MLR [22].

### The Pharmacokinetics of BsB in Mice

**Figure 2:**
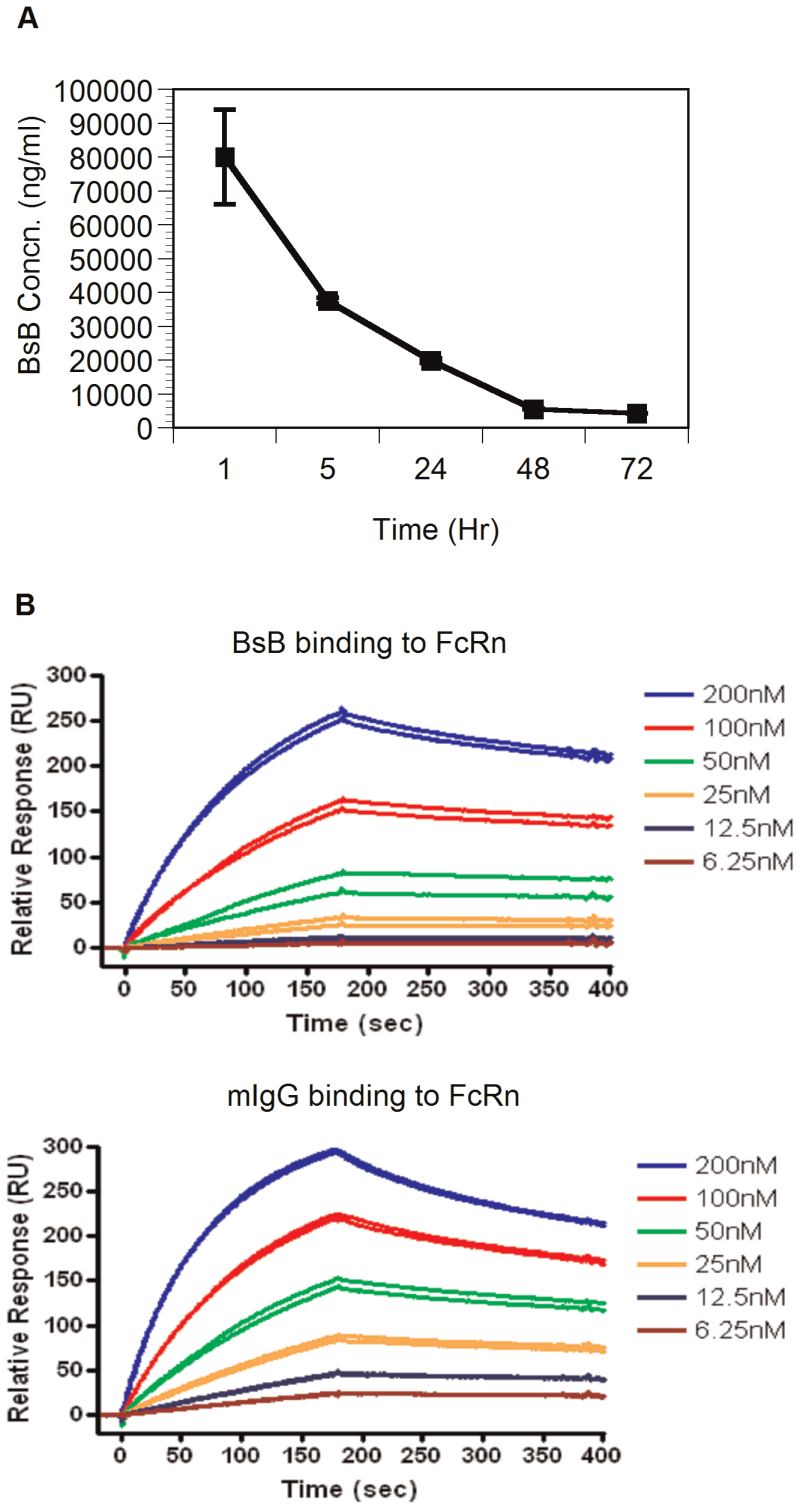
Pharmacokinetics of BsB in vivo and biochemical analysis. (A) The pharmacokinetic profiles of BsB in mice. Normal C57BL/6 mice (n = 5) were dosed intraperitoneally with 20 mg/kg of BsB. Blood samples were collected at the different time points indicated, and the levels of BsB were determined using an ELISA. Data represent mean ± sem. (B) Comparison of the binding of BsB and mouse IgG2a to FcRn. The FcRn were immobilized to a Biacore chip as described in the material and methods. BsB or control mouse IgG2a was loaded onto the chip at various concentrations, and the signals were then recorded.

Prior to testing the therapeutic utility of BsB in animal models of autoimmune diseases, the pharmacokinetic profile of this molecule was determined to help design a dosing regimen in vivo. Intraperitoneal injection of BsB into C57BL/6 mice resulted in a measurable rise in the circulating levels of BsB followed by rapid clearance, with an estimated plasma half-life (t_1/2_) of approximately 12 h (Fig. 2A). This outcome was unexpected because the pharmacokinetic profiles of Fc-containing fusion proteins or antibodies are typically more prolonged. Because the binding of antibodies to the neonatal Fc receptor (FcRn) is primarily responsible for the prolonged half-lives of the Fc-containing fusion proteins [49], we compared the relative abilities of BsB and a control mouse IgG2a to bind the FcRn. Figure 2B shows that the binding characteristics of both proteins to the FcRn were very similar, indicating that a defect in the binding of BsB to FcRn was not likely to be the cause of the rapid clearance of BsB from the circulation.

**Figure 3:**
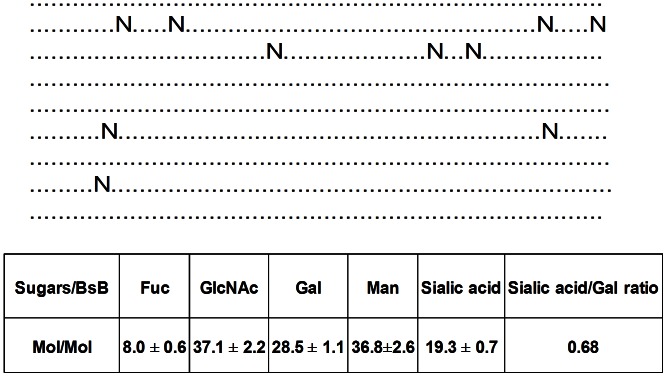
Analysis of asparagine-linked glycosylation on BsB. The amino acid sequence of BsB was submitted to the NetNGlyc 1.0 Server for the prediction of asparagine-linked glycosylation sites. A total of 10 asparagine-linked glycosylation sites (denoted N) were predicted; other amino acids are presented as dots. The monosaccharide composition of BsB was also performed to determine the composition of the glycans. Fucose (Fuc), N-acetylglucosamine (GlcNAc), galactose (Gal), mannose (Man), sialic acid, N-acetylneuraminic acid. A sialic acid:galactose ratio of 0.68 indicates that approximately one third of the galactose residues are available for binding to the ASGPR. Numbers represent mean ± std.

Another potential explanation for the rapid clearance of BsB could be due to its uptake by carbohydrate receptors on non-target cells. Examples of such receptors include the asialoglycoprotein receptor (ASGPR) on hepatocytes [50] and the mannose receptor on macrophages and endothelial cells of the reticuloendothelial system [51]. An analysis of BsB using the NetNGlyc server (http://www.cbs.dtu.dk/services/NetNGlyc/) suggested that BsB has the potential to harbor as many as 10 asparagine-linked oligosaccharide glycans per monomer (Fig. 3). A monosaccharide composition analysis indicated that BsB contained approximately 37 mannose residues, which suggests that all the predicted asparagine-linked glycosylation sites may have been used because each of these asparagine-linked oligosaccharide glycans contains the core-mannose structure with three mannose residues (a total of 30 mannose residues). In addition, it also suggests that a small amount of high-mannose type oligosaccharides may also exist to account for the extra mannose residues. Indeed, significant amounts of under-sialylated tri- and tetra-antennary asparagine-linked, as well as some high-mannose type oligosaccharides were identified by mass spectrometry of permethylated glycans released from the protein (data not shown). This projection is also consistent with BsB’s molecular weight of 100 kDa as suggested by an SDS-PAGE analysis, as opposed to BsB’s calculated weight of 80 kDa. The difference (20 kDa) was likely due to the added presence of oligosaccharides [22]. Moreover, BsB exhibited a ratio of sialic acids to galactose of 0.68 (Fig. 3), indicating that the glycans were incompletely sialylated. Hence, carbohydrate-mediated clearance of BsB by the ASGPR could have been responsible for its rapid clearance from circulation, although other non-identified mechanisms cannot be excluded, such as the binding of BsB to APCs via MHCII. Such binding has been observed in vitro between BsB and APCs (data not shown) although the extent to which this contributed to the rapid clearance of BsB clearance is not known.

### A Short Course of Treatment with BsB Delayed the Onset of Autoimmune Diabetes in NOD Mice in a Late Prevention Model

**Figure 4:**
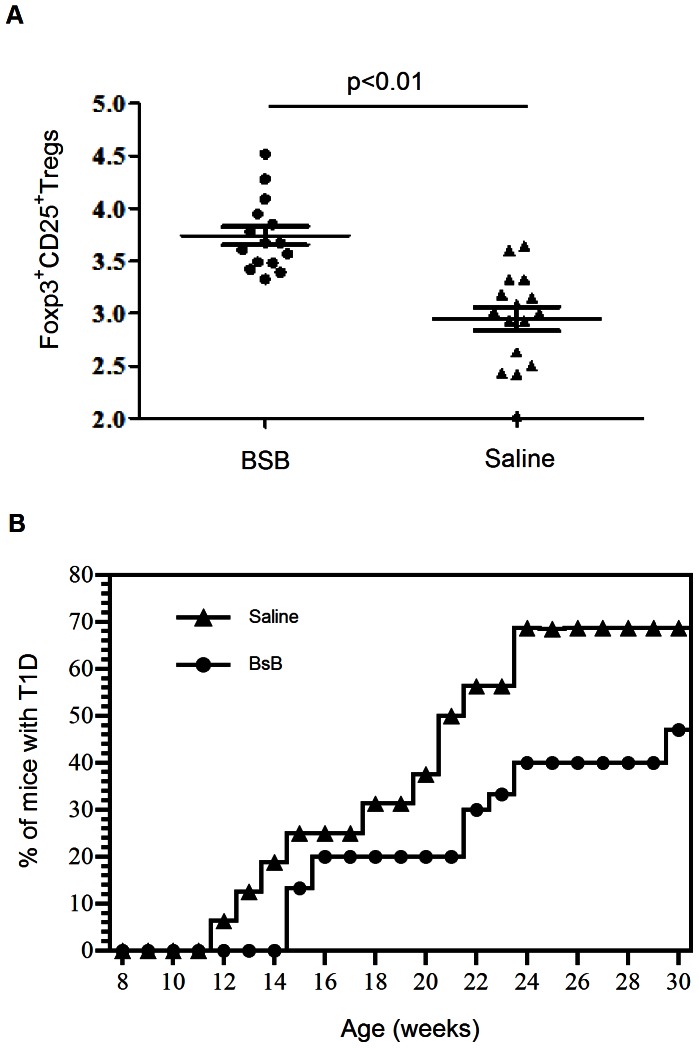
Treatment of NOD mice with BsB modestly delayed the onset of T1D in a late prevention treatment paradigm. **(**A) The levels of Foxp3^+^ Tregs in the blood of BsB-treated (closed circles, n = 15) and saline-treated control mice (closed triangles, n = 14). There was a moderate but significant increase in the number of Tregs in the BsB-treated animals over the number of Tregs noted in the control animals. (B) The cumulative incidences of overt diabetes in animals treated with BsB (filled circles) or saline (filled triangles).

The demonstration that BsB was able to induce Tregs in an antigen-specific manner in vitro prompted us to test whether it could facilitate a similar Treg induction in vivo and thereby treat autoimmune diseases caused by the reaction of autoreactive T cells to autoantigens. We elected to initially test this concept in the NOD mouse model of autoimmune T1D. Bluestone and colleagues [52] have shown that transgenic B cells decorated with surface-linked agonistic CTLA-4 scFv antibodies are able to prevent NOD mice from developing T1D. Moreover, Shieh et al. (2009) reported that surface-linked agonistic CTLA-4 scFv on β-cells also protects NOD mice from developing the disease. Because the EC_50_ of BsB at inducing Tregs in vitro was estimated to be approximately 100 nM and because its circulating half-life was short (t_1/2_ of ∼12 h), we elected to first test BsB in female NOD mice in a late prevention paradigm. The NOD mice were administered BsB over a short interval (every other day during the weekdays for 4 weeks) when the mice were between 9 and 12 weeks of age. At this age, the presence of autoreactive T cells and insulitis are already evident, but the mice have yet to develop overt diabetes. As shown in Figure 4A, the NOD mice treated for 2 weeks with BsB showed a modest but statistically significant increase (25%) in the number of Foxp3^+^ Tregs in the blood compared with the saline-treated controls. However, since we were unable to measure a difference in the number of Tregs after 4 weeks of treatment or at later time points, this increase in the Tregs was transient. A similar transient increase in Tregs in the lymphoid organs was previously noted following treatment of NOD mice with an anti-CD3 antibody [53]. It is possible that the BsB-induced Tregs reverted to Foxp3^−^ T cells after the cessation of treatment. More likely, the Tregs may have been recruited by specific target tissues (e.g., the pancreas) to execute their function. Regardless, this short course of treatment with BsB in a late prevention treatment paradigm appeared to modestly delay the onset of disease and decreased the number of mice presenting with overt T1D (Fig. 4B).

### A Longer Course and Earlier Treatment with BsB Significantly Delayed the Onset and Reduced the Incidence of Autoimmune Diabetes in NOD Mice

**Figure 5:**
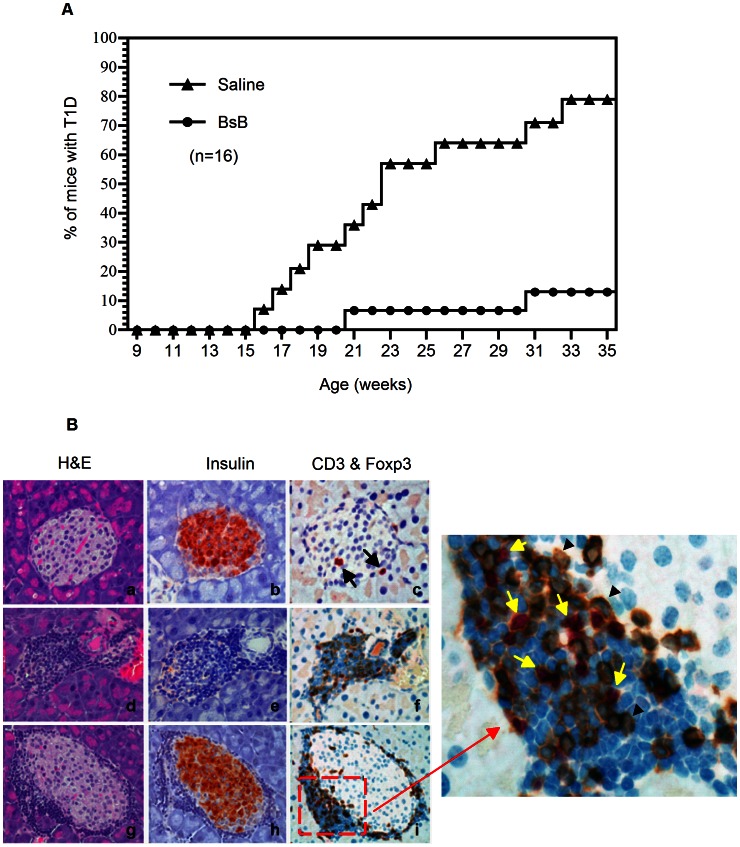
Longer-term treatment of NOD mice with BsB significantly delayed the onset of T1D in NOD mice. (A) The cumulative incidences of overt diabetes in BsB-treated (n = 16) and untreated mice (n = 16). BsB treatment significantly reduced the incidence of T1D compared with mice treated with saline (*p*<0.01). (B) A histopathological analysis of pancreatic tissues from animals treated with saline or BsB. Panels a through c represent the sections from saline-treated mice that remained non-diabetic with H&E, an antibody to insulin (pink), or anti-CD3 (brown) and Foxp3 (pink), respectively. Similar observations were noted in BsB-treated NOD mice that remained disease-free. No evidence of infiltration or insulitis was noted in any of the sections; a few Foxp3^+^ Tregs may be present (arrows in panel c). Panels d through f represent the pancreatic sections from diabetic NOD animals. Invasive insulitis was clearly evident, and the insulin-producing β-cells were completely destroyed (e). Several CD3^+^ T cell infiltrations were also detected along with a few Tregs and many non-T cell leukocytes with blue nuclei (f). Panels g through i show that the islets of BsB treated animals that remained non-diabetic but exhibited characteristic peri-insulitis. Leukocyte infiltrations were noted but were restricted to the periphery of the islets. Moreover, there was no notable destruction of the insulin-producing β-cells. Most of the leukocytes at the periphery were non-T cells (with blue nuclei). The enlarged inset (panel j, represents the red square in i) indicated Foxp3^+^ Tregs (yellow arrow head) were intermixed with other CD3^+^ T cells and non-T cell leukocytes (with blue nuclei) at the periphery of islets. The images were acquired with a 40× objective; the inset was acquired with a 60× objective, which was then further enlarged 3× digitally.

The modest response in the above late treatment paradigm might have been due to the presence of active insulitis in the 9-week-old NOD mice prior to the commencement of therapy or the short course itself. An inflammatory milieu has been shown to favor the conversion of T cells to Th17 cells and to suppress the conversion of T cells to Tregs. Inflammatory cytokines, such as IL-6 or IL-4, have also been shown to inhibit Treg conversion and to promote the loss of Foxp3^+^ expression in Tregs [54]–[56]. Alternatively, the modest response could be due to the deployment of a relatively short course of treatment, the moderate potency of BsB at inducing the production of Tregs (EC50 at >100 nM in vitro), and the short circulating half-life of BsB, which may have limited its exposure. As the potency and circulating half-life of BsB are intrinsic to the molecule and therefore not amenable to facile change, we elected to test the merits of a longer course of treatment in the context of an early prevention model. To this end, NOD mice were treated with BsB for 10 weeks, beginning when the mice were 4 weeks of age. As shown in Figure 5A, the NOD mice treated for 10 weeks with BsB exhibited a significant delay in the onset of T1D. Importantly, by 35 weeks of age, only 13% of the BsB-treated NOD mice had developed T1D, compared to 80% of the saline-treated controls. Thus, the extended treatment of NOD mice with BsB and the early treatment appear to have protected the animals from developing autoimmune diabetes.

At the conclusion of the study (when the mice were 35 weeks old), the animals were sacrificed, and their pancreata were collected for histopathological analysis. Adjacent serial sections were stained with H&E for a general assessment of the islets for insulits, probed with an anti-insulin antibody to detect the presence of insulin in the β-cells, and double stained with anti-CD3 and anti-Foxp3 antibodies to locate T cells and Tregs.

Analysis of the histopathology and immunohistochemical stains of sections from saline-treated NOD mice revealed three phenotypes. The first was of a small number (19%) of untreated animals that did not develop disease at 35 weeks of age, which was due to the heterogeneity of T1D onset in the NOD strain. Analysis of their islets showed that the β-cells were intact and producing insulin, with no obvious evidence of lymphocytic infiltration or insulitis (Fig. 5B, panels a–c). A few Foxp3^+^ Treg cells were present in the islets of these mice (arrows in panel c). A second phenotype was noted in the islets of diabetic NOD mice. These animals exhibited the presence of severe insulitis (Fig. 5B, panel d) and the complete destruction of the β-cells as indicated by a lack of insulin staining (panel e). In addition to the presence of CD3^+^ T cells and Foxp3^+^ Tregs, large numbers of non-T-cell lymphocytes were also evident (Fig. 5B, panel f). The third was noted in the islets of a small number of diabetic animals that were determined to be free of insulitis; nevertheless, the islets were also completely devoid of insulin staining (Figure S1, panels a–d), indicating that the β-cells in these islets were destroyed or functionally inactive. The findings in the third phenotype are not without precedence as a lack of correlation between insulitis and T1D in the NOD mouse has been documented previously [65], [66]. Thus, the insulitis-free but functionally inactive islets noted here in the diabetic animals are reminiscent of the post-insulitis pseudo-islets mentioned previously [66]. It is possible that this was due in part to the late timing of sacrifice of these animals (at 35 weeks of age), which is past the optimal time for insulitis assessment.

Similar histopathological findings were observed in the islets of animals treated with BsB. The two animals that developed diabetes had severe insulitis and lacked insulin staining in the islets (similar to phenotype 2 noted in the control animals, data not shown). Interestingly, in the islets of BsB-treated animals that remained non-diabetic, evidence of non-insulitis (similar to phenotype 1 described above for control animals) and moderate to severe insulitis were noted (Fig. 5B, panel g, and Figure S1, panels e, g and i). However, in both cases, the β-cells were still well preserved and producing insulin (Fig. 5B, panel h, and Figure S1, panels f, h, j). Staining with antibodies indicated that the cells at the periphery of the islets were comprised primarily of CD3^+^ T cells and Tregs (Fig. 5B, panel i). An enlargement of a section of the image (the red square in Fig. 5B, panel i) clearly revealed the presence of numerous Foxp3^+^ Tregs (the yellow arrows in Fig. 5B, panel j) that were interspersed with non-Foxp3^+^ but CD3^+^ T cells (the black arrowheads in Fig. 5B, panel j) and non-T-cell mononucleocytes (with blue nuclei). The presence of insulitis has been noted in young (4- to 10-week-old) NOD mice [42] and in older mice treated with other efficacious therapeutic agents that delayed or reversed newly onset T1D in NOD mice [57]–[60]. Our data indicated that BsB-treatment was also effective at preserving insulin-producing β-cells but not prevent the development of insulitis at 35 weeks of age.

Tregs in the blood were also monitored at various time points after two weeks of BsB treatment. In contrast to the late prevention paradigm described above in Fig. 4, no noticeable increase in Tregs was observed in early treatment setting (data not shown). This observation is similar to the result of a separate early prevention study (Figure S2) where we also included mouse IgG2a as an isotype control. Accordingly, although we would like to speculate on the possibility that this protection was mediated, at least in part, by the de novo and possibly in situ induction of Tregs and the production of IL-10 and TGF-β, a definitive confirmation is needed to substantiate this hypothesis.

## Discussion

In this study, we showed that crosslinking CTLA-4 and TCR via MHCII using a novel bispecific fusion protein (BsB) efficiently induced the production of antigen-specific Tregs and the anti-inflammatory cytokines, IL-10 and TGF-β in vitro. Previous studies showing that Tregs are critical for conferring immune tolerance and that antigen-specific Tregs are more efficacious in animal models of autoimmune diseases encouraged us to further evaluate BsB in animal models of autoimmune diseases, such as T1D. We hypothesized that if BsB promoted the induction of antigen-specific Tregs during the early phase of activation of autoreactive T cells in NOD mice, this process might delay the onset or halt the progression of the disease by converting the autoreactive T cells that are undergoing activation into Tregs.

Despite the fact that our proof-of-concept BsB exhibits a modest potency (due to its moderate affinities for the MHCII and CTLA-4) and a short circulating half-life (which limited its exposure), a short course of treatment reproducibly delayed the onset of T1D in NOD mice treated at an early age (between 4 to 6 weeks of age, Figure S2) and when they were older (between 9 to 12 weeks of age), albeit with modest benefits (Fig. 4). A longer course of treatment (10 weeks) of NOD mice (between 4 and 13 weeks of age) with BsB significantly delayed the onset of the disease and reduced the incidence of animals developing T1D (Fig. 5). This benefit was likely imparted by the de novo generation of induced Tregs that were either produced locally (e.g., in the pancreas or pancreatic-draining lymph nodes) or distally that were then recruited to the pancreas to protect the islets from destruction by autoreactive T cells and other non-T cell leukocytes. This was evidenced by the presence of many Foxp3^+^ Tregs at the periphery of the islets in the pancreatic tissues from 35-week-old BsB-treated mice that remained non-diabetic (Fig. 5B). Unarguably, determining the antigen specificities of these Tregs would shed more light on the mechanism of action of BsB in vivo. Similarly, quantitation of Tregs in the pancreata and draining lymph nodes of the pancreata may also offer valuable information. However, these were not investigated here due to technical limitations.

A modest but statistically significant increase in the number of Foxp3^+^ Tregs was detected in the blood of the BsB-treated animals (treated at 9 to 12 weeks of age) compared with the untreated controls. This increase was not evident in the mice that started treatment at a younger age (4 weeks old). The reason for this discrepancy remains unclear but might be due to the fact that there were more autoreactive T cells undergoing activation in the 9-week-old mice than in the 4-week-old mice; thus, the low levels of autoreactive T cells in the 4-week-old mice might have precluded the detection of induced Tregs beyond those in the existing milieu. Additionally, the increase of Tregs in the blood was transient. A similar observation was noted in animals subjected to anti-CD3 therapy [53]. It is possible that the induced Tregs were unstable and lost their expression of Foxp3. More conceivable, the Tregs were recruited from the circulation to the affected target tissues. The appearance of insulitis is typically observed in the pancreas of NOD mice between 4 and 9 weeks of age. If uncontrolled, more severe insulitis ensues, leading to the complete destruction of β-cells and the development of overt diabetes between 12 and 35 weeks of age in ∼80% of female mice. In this study, however, the insulitis status in the control animals and the BsB-treated animals were comparable (not shown), suggesting that BsB may have protected β-cell function without significantly reducing the severity of insulitis. In contrast, the lack of insulitis or mild insulitis noted in some of the diabetic animals in the control group was not associated with functionally active β-cells in the islets (Figure S1). Although we have not defined the exact mechanism by which BsB worked in NOD mice, our immunohistochemical findings (Fig. 5B) are most akin to those reported by Lee et al. [62], suggesting the involvement of induced Tregs in preventing T1D onset. Lee et al. [62] showed that the transfer of diabetogenic CD4^+^CD25^−^ BDC2.5 T cells depleted of CD4^+^CD25^+^ Tregs into female NOD/SCID mice expedited the development of invasive insulitis compared with mice administered total CD4^+^ T cells containing CD4^+^CD25^+^ Tregs. The invasive insulitis was largely dominated by the infiltration of dendritic cells (DCs) rather than by BDC2.5 T cells per se. The authors surmised from their study that the Tregs regulated the invasiveness of the DCs into the islets by modulating, at least in part, the chemotaxis of the DCs in response to the chemokines CCL19 and CCL21 secreted by the islets. Similarly, Sarween et al. [63] also reported that Tregs potently suppress pathogenic T cell infiltration into islets by inhibiting IFN-γ induced chemokine receptor CXCR3 expression. Regardless, there are reports of different therapeutic interventions capable of delaying or preventing disease in NOD mice via different mechanisms [61], hence, BsB may be acting through a distintive mechanism other than Tregs.

The demonstration of the combined production of IL-10, TGF-β and Tregs in response to treatment with BsB in vitro as well as efficacy in the NOD mouse model of T1D encourages further evaluation of this novel therapeutic concept. BsB also offers additional advantages over other immune modulators in that it does not affect resting T cells or other lymphocytes. The numbers and percentages of CD4^+^ T cells and CD19^+^ B cells in the periphery remained the same in all of our NOD studies (data not shown). Going forward, it will be important to determine whether this approach is effective not only in delaying or halting disease progression but also in reversing newly-onset T1D. The facilitation of these studies will require the development of BsB variants that are more potent (e.g., a high affinity bispecific antibody recognizing MHCII and being agonistic to CTLA-4) and that harbor a more favorable pharmacokinetic profile. Additionally, more sophisticated analytical tools are needed to demonstrate that the induction of antigen-specific Tregs was achieved in response to treatment by BsB in vivo. If successful, this concept may also be applied towards the management of other immune-mediated diseases.

## Materials and Methods

### Animals

Female wild-type C57BL/6 (H-2^b^), BALB/c(H-2^d^), transgenic OT-II mice expressing the mouse α-chain and β-chain T cell receptor specific for chicken ovalbumin 323–339(Ova_323–339_) on the C57BL/6 genetic background, and female NOD (NOD/LtJ) mice were purchased from The Jackson Laboratory. The animals were maintained in a pathogen-free facility, and the studies were conducted in accordance with the guidelines issued by the U.S. Department of Health and Human Services (NIH Publication No 86–23) and approved by Genzyme’s Institutional Animal Care and Use committee.

### Antibodies and Reagents

The functional grade or fluorescently labeled anti-mouse CD3 (clone 145-2C11), CD25, insulin and Foxp3^+^ antibodies were purchased from eBioscience or BD Biosciences. The murine CTLA-4-Fc and human CTLA-4Ig (Orencia) were purchased from R&D Systems, Inc. and Bristol-Myers Squibb, respectively. The mouse IgG2a isotype control was obtained from BioXCell Inc. CFSE, ultralow Ig fetal bovine serum (FBS), and other cell culture media were from Invitrogen. The chicken Ova_323–339_ peptide was obtained from New England Peptide.

### The Construction and Production of the Bispecific Fusion Protein BsB

The construction and expression of BsB, which was composed of the extracellular domains of CD80w88a and LAG-3 as well as the Fc of mouse IgG2a (CD80wa-LAG-3-Fc, BsB), were described previously [22].

### Biacore Assays and Monosaccharide Composition Analysis

The Biacore was used to compare the binding of BsB and mIgG2a to the mouse neonatal Fc receptor (FcRn). Briefly, a CM5 chip was immobilized with approximately 1430 RU of mouse FcRn-HPC4 using amine chemistry. Each sample was serially diluted 1∶2 to final concentrations between 200 and 6.25 nM in PBSP (PBS with 0.005% Surfactant P-20), pH 6.0, and injected for 3 min in duplicate, followed by a 3 min wash with dissociation buffer. The surface was regenerated with 10 mM sodium borate and 1 M NaCl, pH 8.5. The carbohydrate monosaccharide composition of BsB was analyzed according to the protocol described by Zhou et al. [64].

### The Isolation of Naïve T cells

Naive T cells from the spleens and lymph nodes of 8- to 12-week-old female OT-II mice were purified by magnetic separation followed by fluorescence-activated cell sorting. Mononuclear splenic cell suspension was labeled using Miltenyi’s negative selection CD4^+^ T cell magnetic isolation kit according to manufacturer’s instructions. Labeled (CD4^−^ cells) and unlabeled (CD4^+^ cells) were separated using Miltenyi magnetic columns. Cells were then stained with a live/dead discriminant stain, anti-CD4, anti-CD25, anti-CD44 and anti-CD62L antibodies (eBioscience). The resulting cell suspension was sorted for live CD4^+^CD25^low^CD62L^high^CD44^low^ phenotype. All resulting purified cell populations were >98% pure as assessed by flow cytometry.

### The Antigen-specific Treg Induction Assay

The assays in an allogenic MLR setting were performed as previously reported [22]. For antigen-specific T cell activation, 10^5^ naïve OT-II T cells were mixed in round-bottom 96-well plates in duplicates with 10^5^ irradiated syngeneic APCs in the presence of Ova_323–329_ at 0.5 µg/ml and 1 µg/ml soluble anti-CD28 (clone 37.51, eBioscience). The test constructs, mouse IgG2a, or mouse CTLA-4Ig were added to the cultured cells at saturating concentrations of 100 µg/ml. The cells were cultured for 5 days to induce the production of Tregs and analyzed by flow cytometry. The media were collected for the analysis of IL-2, IL-10 and TGF-β using ELISA kits per the manufacturer’s instructions. To assess the proliferation of the T cells, purified naïve OT-II T cells were labeled with 5 µM CFSE for 5 min at 37°C. The cells were then washed to remove unbound CFSE and used in Treg induction assays as described above. The cells were cultured for 5 days to allow them to divide before being analyzed by flow cytometry. To detect Foxp3^+^ in the T cells, the cells were stained for surface markers, as described above, and then permeabilized with Fix/Perm buffer (eBioscience) and stained with anti-Foxp3 antibody (clone FJK-16s, eBioscience).

### The Pharmacokinetics Measurements of BsB in Mice

The pharmacokinetics of BsB was determined in 8-week-old C57BL/6 mice. Twenty milligrams per kilogram of BsB was administered into the mice by intraperitoneal injection. The blood was collected by saphenous vein bleeding at 1 h, 5 h, 24 h, 48 h, and 72 h after administration. The levels of BsB at each time point were measured using an ELISA assay. Briefly, 100 µl (1 µg/ml) of an anti-mouse CD80 antibody in PBS was coated onto 96-well plates and incubated overnight at 4°C. The plates were blocked with 5% fetal bovine serum for 1 h, after which they were washed four times with PBS. One hundred µl of blood samples at various dilutions were then added into the wells. The plates were incubated for 2 h with gentle shaking at room temperature and washed four times with PBS. A biotinylated anti-mouse LAG-3 antibody (1 µg/ml) was added and incubated for 2 h. The plates were washed four times with PBS, after which streptavidin-HRP was added. After 30 min, the plates were washed six times with PBS and developed for colorimetric measuring. Purified BsB diluted in the assay diluent at various concentrations was used as standards.

### Treatment of NOD Mice with BsB

For the short-course late prevention model, female NOD mice at 9 weeks of age were treated for 4 weeks with saline or 20 mg/kg BsB. For the short-course early prevention study, female NOD mice at 4 weeks of age were treated with saline, 20 mg/kg mouse IgG2a, 10 mg/kg human CTLA-4Ig (Orencia), or 20 mg/kg BsB. For the longer-course early treatment model, female NOD mice were treated with saline or BsB at 20 mg/kg for 10 weeks, starting at age of 4 weeks to 13 weeks of age. All treatments were administered three times per week every other day during the weekdays by intraperitoneal injection. The non-fasting blood glucose levels were monitored weekly starting at 8 weeks of age. The mice were deemed to be diabetic when their glucose readings were greater than 300 mg/dL for three consecutive readings. Mouse IgG2a isotype was included as a negative control in the early prevention studies with no difference on T1D incidence as compared to saline (Figure S2). The impact of BsB treatment on general T cells and B cells in the peripheral blood were monitored by staining with fluorescently labeled anti-CD3 and anti-CD19 antibodies followed by FACS analysis. The Foxp3^+^ Tregs in the peripheral blood were examined by flow cytometry after 2 weeks of treatment. Briefly, 50 µl of whole blood was blocked with unlabeled anti-FcγRIIb and FcγRIII (clone 93, eBioscience) for 20 min. The cells were subsequently stained with fluorescently labeled anti-CD4 antibody for 30 min and then washed. The red blood cells were lysed using FACS Lysing solution (BD Biosciences) for 5 min. After washing, the cells were fixed, permeabilized and stained with a FITC-labeled anti-Foxp3 antibody for 30 min as described above. The pancreata were fixed in neutral buffer formalin.

### Histopathology and Immunohistochemistry

Pancreatic tissues were fixed in 10% neutral buffered formalin, routinely processed, paraffin-embedded, sectioned and stained with hematoxylin and eosin for assessment of insulitis. The neutral buffer formalin-fixed pancreata were stained for CD3 and Foxp3^+^ cells using an automated processor. The tissue sections were dewaxed using xylene-ethanol, and the antigens retrieved by incubating for 25 min in citrate buffer and then blocked with serum. The slides were incubated with an anti-CD3 antibody for 45 min, followed by a goat anti-rabbit horseradish peroxidase polymer for 20 min. The chromogen visualization of CD3 was obtained by incubating with 3,3′-diaminobenzidine tetrahydrochloride for 2 to 4 min. To detect Foxp3^+^, the sections were re-blocked with serum followed by exposure to an anti-Foxp3 antibody for 45 min. The slides were then incubated with a rabbit anti-rat IgG antibody for 30 min followed by a goat anti-rabbit alkaline phosphatase polymer. The chromogen visualization was performed using Fast Red for 10 min. The tissue sections were counterstained using hematoxylin for 2 min and washed 3 times with 0.05% Tween-20/Tris buffered saline between steps. The adjacent serial sections were stained using an anti-insulin antibody as described above. The pictures were taken using a Nikon Eclipse E800 fluorescent microscope with an attached digital camera from Diagnostic Inc., and images were acquired using the Spot Advanced software.

### Statistical Analysis

Cumulative incidences of NOD mice presenting with T1D and hyperglycemia following treatment with BsB or controls were compared using the log-rank (Cox-Mantel) test in Prism 5 (GraphPad). A value of *p*<0.05 was considered to be statistically significant.

## Supporting Information

Figure S1 **Histopathological analysis of islets from surviving animals in the long-term BsB-treated study.**
(DOCX)

Figure S2 **Treatment of NOD mice with BsB delayed the onset of T1D in an early prevention treatment paradigm.**
(DOCX)
