# Supplementary material for: A Bispecific Protein Capable of Engaging CTLA-4 and MHCII Protects Non-Obese Diabetic Mice from Autoimmune Diabetes
Source: PLoS One. 2013 May 21;8(5):e63530. doi: 10.1371/journal.pone.0063530 (PMC3660570; doi:10.1371/journal.pone.0063530)

Figure S1. Histopathological analysis of islets from surviving animals in the long-term BsB-treated study. Representative images of pancreatic islets of NOD mice demonstrated the variability of insulitis lesions. Sections of pancreas were stained with H&E (left panels) and labeled with an anti-insulin antibody (right panels). Panels (a-d) were representatives from different animals that became diabetic during the course of the study. The depicted islets were insulitis-free, but lacked insulin reactivity. Panels (e-j) were from different BsB-treated animals that remained non-diabetic. These islets had varying degrees of moderate insulitis; however all showed preservation of insulin immunoreactivity (brown staining) in remaining β-cells.

Figure S1.


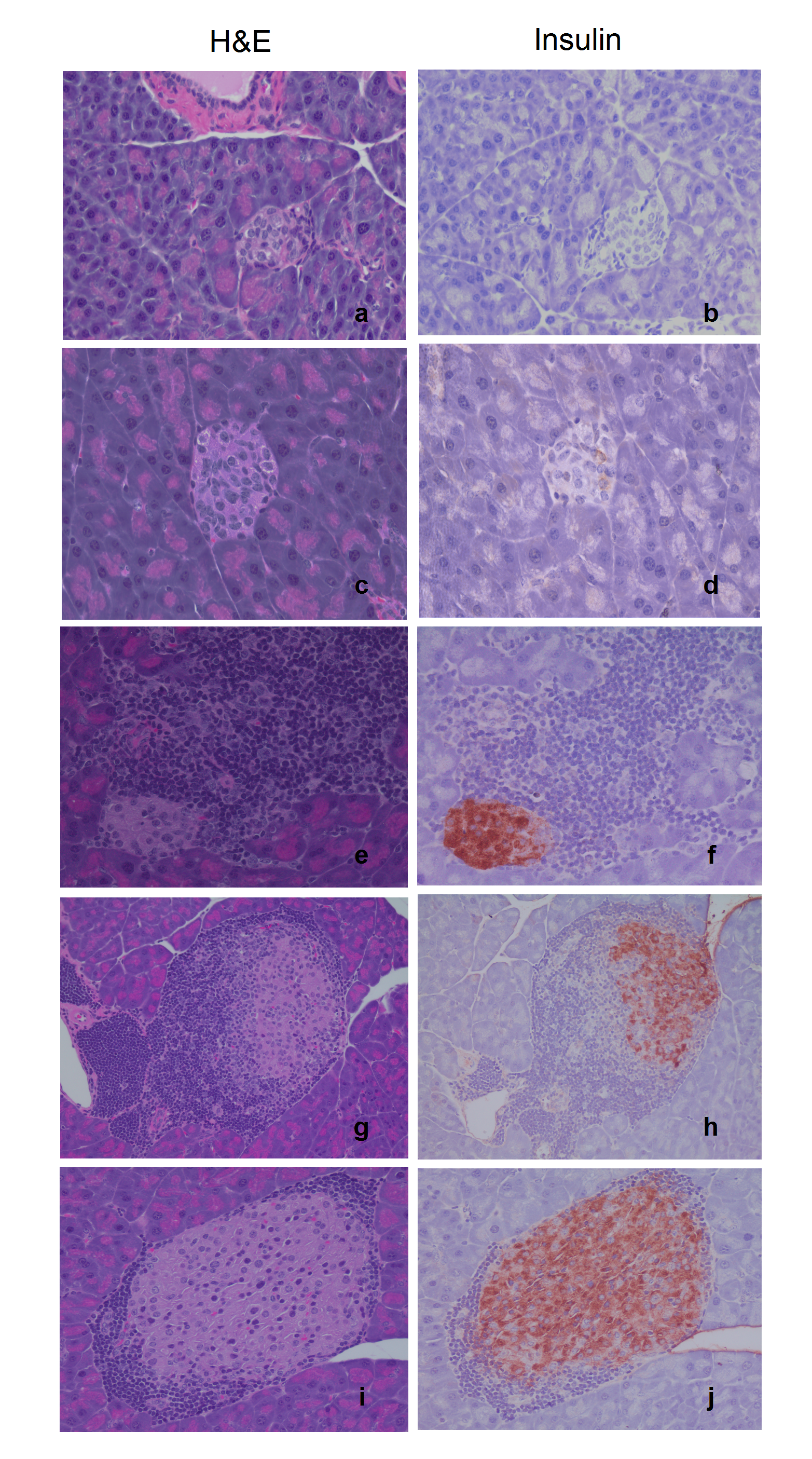

Supplement: Figure S1 — Histopathological analysis of islets from surviving animals in the long-term BsB-treated study. (DOCX) [file pone.0063530.s001.docx]
