# Supplementary material for: A Bispecific Protein Capable of Engaging CTLA-4 and MHCII Protects Non-Obese Diabetic Mice from Autoimmune Diabetes
Source: PLoS One. 2013 May 21;8(5):e63530. doi: 10.1371/journal.pone.0063530 (PMC3660570; doi:10.1371/journal.pone.0063530)

Figure S2. Treatment of NOD mice with BsB delayed the onset of T1D in an early prevention treatment paradigm. (A) The levels of Foxp3^+^ Tregs in the blood of mice treated with BsB (closed circles), saline (closed triangles), CTLA-4Ig (closed squares) and mouse IgG2a (open squares) are shown. No increase in the number of Foxp3^+^ Tregs was detected after two weeks of treatment with BsB compared with saline or mIgG2a-treated controls. However, treatment with CTLA-4Ig resulted in a statistically significant decrease in the number of Foxp3^+^ Tregs in the blood, likely due to blockade of the co-stimulatory pathway required for Treg homeostasis. This reduction of Tregs also correlated with an exacerbated onset of T1D. Here, only 10 of 16 animals in each group were analyzed for Foxp3^+^ Tregs in the blood. (B) The cumulative incidences of overt diabetes in animals treated with BsB or controls. BsB treatment resulted in a significant delay in the onset of T1D compared with the saline or mouse IgG2a-treated control groups before 24 weeks of age (p=0.04). However, no significant difference between the groups was noted at the end of the study. The data represent one (n=16) of two separate studies with similar results. Also please note that there was no significant difference in the course of T1D onset between the saline and mouse IgG2a treated groups.

Figure S2.


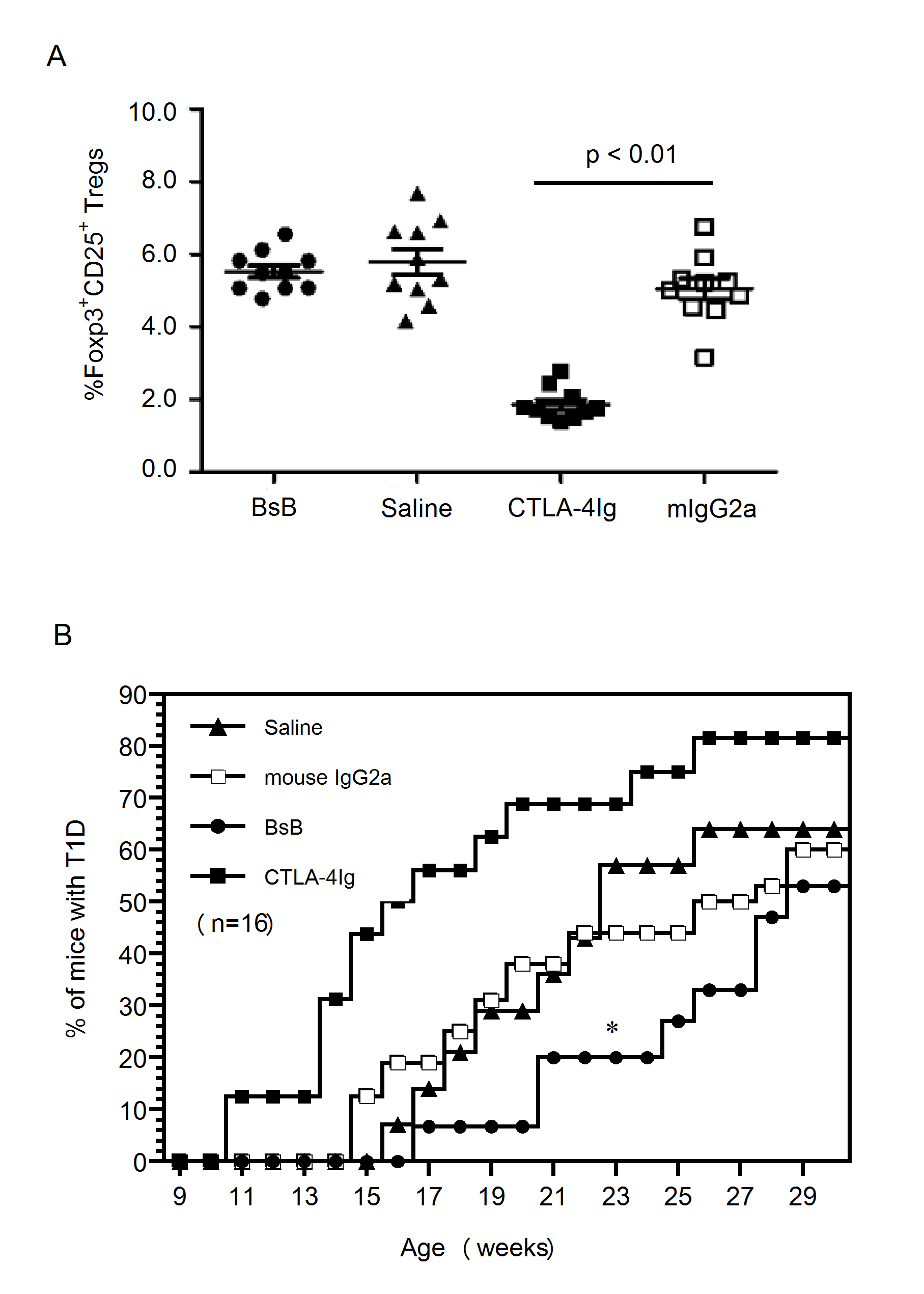

Supplement: Figure S2 — Treatment of NOD mice with BsB delayed the onset of T1D in an early prevention treatment paradigm. (DOCX) [file pone.0063530.s002.docx]
